# Evaluation of the Role of Soybean Lecithin, Egg Yolk Lecithin, and Krill Oil in Promoting Ovarian Development in the Female Redclaw Crayfish *Cherax quadricarinatus*

**DOI:** 10.1155/2023/6925320

**Published:** 2023-01-12

**Authors:** Chang Xu, Xiaolong Yang, Zhenye Liang, Zongzheng Jiang, Hu Chen, Fenglu Han, Yongyi Jia, Erchao Li

**Affiliations:** ^1^Key Laboratory of Tropical Hydrobiology and Biotechnology of Hainan Province, Hainan Aquaculture Breeding Engineering Research Center, College of Marine Sciences, Hainan University, Haikou, Hainan 570228, China; ^2^Agriculture Ministry Key Laboratory of Healthy Freshwater Aquaculture, Key Laboratory of Fish Health and Nutrition of Zhejiang Province, Key Laboratory of Freshwater Aquaculture Genetics and Breeding of Zhejiang Province, Zhejiang Institute of Freshwater Fisheries, Huzhou, China

## Abstract

The optimal supplementation of lipid nutrients in the diet showed crucial physiological functions in gonadal development and maturation in adult female aquatic animals. Four isonitrogenous and isolipidic diets with no extra lecithin supplementation (control), 2% soybean lecithin (SL), egg yolk lecithin (EL), or krill oil (KO) supplementation were formulated for *Cherax quadricarinatus* (72.32 ± 3.58 g). Ovary development and physiological characteristics of crayfish were evaluated after a 10-week feeding trial. The results indicated that SL, EL, or KO supplementation all significantly increased the gonadosomatic index, especially in the KO group. Crayfish fed the diet with SL showed the highest hepatosomatic index compared with those fed the other experimental diets. KO was more efficient than SL and EL in promoting triacylglycerol and cholesterol deposition in the ovary and hepatopancreas but also showed the lowest concentration of low-density lipoprotein cholesterol in the serum. KO significantly increased yolk granule deposition and accelerated oocyte maturation than other experimental groups. Furthermore, dietary phospholipids significantly enhanced the gonad-stimulating hormone concentration in the ovary and reduced the secretion of gonad-inhibiting hormones in the eyestalk. KO supplementation also significantly improved organic antioxidant capacity. From the ovarian lipidomics results, phosphatidylcholine and phosphatidylethanolamine are two main glycerophospholipids that respond to different dietary phospholipids. Polyunsaturated fatty acids (especially C18:2n-6, C18:3n-3, C20:4n-6, C20:5n-3, and C22:6n-3) were pivotal participants during ovarian development of crayfish regardless of lipid type. Combined with the ovarian transcriptome, the best positive function of KO was due to activated steroid hormone biosynthesis, sphingolipid signaling, retinol metabolism, lipolysis, starch and sucrose metabolism, vitamin digestion and absorption, and pancreatic secretion. As a consequence, dietary supplementation with SL, EL, or KO all improved the ovarian development quality of *C. quadricarinatus*, especially KO, which was the optimum choice for promoting ovary development in adult female *C. quadricarinatus*.

## 1. Introduction

Unlike viviparous animals, oviparous animals' embryos develop, mature, and spawn independently outside the female body and rely mainly on the energy and nutrients supplemented by the yolk within the egg [1]. From oogonia to oocyte maturation, vitellogenin (Vg) is synthesized and accumulates in oocytes. In insects, Vg is extravaginally synthesized by the female fat body in response to juvenile hormones (JH) and subsequently transported to oocytes by receptor-mediated endocytosis through the hemolymph [2]. Crustaceans, like insects, also rely on exogenous Vg synthesis by a series of hormones, including gonad-inhibiting hormone (GIH), methyl farnesoate (MF), estradiol (E2), and progesterone, particularly in the hepatopancreas [3, 4]. The hepatopancreas of crustaceans' functions in homology to the fat body of insects and the liver or liver-pancreas of vertebrates. Lipid substrates are deposited in the blister-like cells of the hepatopancreas [4]. The development of oocytes has been shown to be accompanied by massive lipid transfer from the endogenous hepatopancreas to the ovary in several crustaceans for both energy supplementation and Vg transport [5, 6].

Lipids play a number of roles in animal life histories, particularly in the female reproductive stage. Lipid accumulation in the hepatopancreas is insufficient to support the quick and high demand for lipids in ovarian development, and excess lipid nutrient ingestion is still needed. Neutral lipids, such as triacylglycerols (TGs), are the main source of storage energy for gonadal development and can accumulate in the ovary as lipid droplets [7]. Compared to TGs, polar lipids, including glycerophospholipids (GPLs) and sphingolipids, are more physiologically active molecules. GPLs consist of fatty acids (FAs), a hydrophilic residue and a phosphate group. FAs can be esterified to the glycerol backbone in GPLs. As the two most abundant GPLs, phosphatidylcholine (PC) and phosphatidylethanolamine (PE) participate in several physiological processes including signaling transport, the activity of membrane-bound enzymes, lipid transport through the hemolymph, and even the anti-inflammatory response in organisms [8]. For embryonic development, sphingolipids are also essential for neurogenesis [9]. Moreover, phospholipid ingestion and absorption are also potential approaches to supply oocytes with polyunsaturated fatty acids (PUFAs) [10]. Previous research indicated that low-level supplementation of lipids in a broodstock diet can diminish egg quality [11]. Conversely, optimum lipid supplementation in the diet can significantly improve the vitellogenesis and gonad development of crayfish [12, 13]. Research has indicated that phospholipid supplementation in diets can also significantly improve gonadal development and egg quality in other crustaceans including shrimp, crab, and macrobrachium [14, 15]. Therefore, the regulation of dietary lipid nutrients is an effective method to improve reproductive performance in female crustaceans.

Crayfish are a taxonomic group summarized as freshwater crustaceans that contain both critically endangered endemic species and highly attractive invasive species [16]. The redclaw crayfish *Cherax quadricarinatus* is almost the largest freshwater decapod and is native to northern Australia and southern New Guinea. From the perspective of aquaculture and commercial development, *C. quadricarinatus* can be a crucial species due to its high tolerance range of ambient temperature, salinity, pH, and dissolved oxygen [17, 18]. Crayfish eggs have the biological characteristics of larger fertilization (2.2-2.3 mm in diameter) and attachment in pleopods [7]. Meanwhile, this species can spawn multiple times (three to five times within a year) if the conditions are appropriate [19]. After the first spawning, they can reach secondary gonadal maturity and continue to mate and spawn in a month. The lipid nutritional composition in compound feed can be a critical ingredient for gonadal development due to the low biosynthesis rate of phospholipids and PUFAs in crayfish. Sufficient and appropriate lipid nutrition supplementation is an effective method to guarantee rapid gonadal development and improve fertilized egg quality. When supplemented with ≥2% dietary soybean lecithin, crayfish showed significantly higher gonadosomatic index [20]. However, animal phospholipids (egg yolk phospholipid and krill oil) appear to have more pronounced gonadotropic effects in *Litopenaeus vannamei* and *Eriocheir sinensis* [21, 22]. To date, no sources of phospholipids other than soybean lecithin have been evaluated in *C. quadricarinatus*. Therefore, the purpose of this paper is to explore the optimum choice of three different sources of lipids for preadult female *C. quadricarinatus*.

The present study was carried out in female *C. quadricarinatus* to investigate the nutritional function of soybean lecithin, egg yolk lecithin, and krill oil on gonadal development, oocyte quality, lipid deposition, hormone secretion, and antioxidant health during reproduction. The present results can provide a theoretical foundation for the further improvement of nutritional enhancement formulas of compound feed for adult female crayfish.

## 2. Materials and Methods

### 2.1. Animal Ethics

Experimental procedures and designs in this present study were approved by the Hainan University Institutional Animal Use and Care Committee, Haikou, China (HNUAUCC-2020-00004).

### 2.2. Experimental Diets

Four isonitrogenous and isolipidic practical experimental diets were formulated containing 0% phospholipid supplement (control), 2% soybean lecithin (SL), egg yolk lecithin (EL), or krill oil (KO) (dry matter). The ingredients and proximate composition of the four experimental diets are presented in Table 1. Dietary protein sources were mainly consisting of fish meal, soybean meal, and cottonseed meal (approximately 43.5% crude protein). Dietary lipid sources were mainly consisting of soybean oil and cholesterol (crude lipid content is 14.3% approximately). To avoid lipid peroxidation in diets, butylated hydroxytoluene (BHT) were added at the concentration of 0.05‰. Three protein source ingredients were ground and sifted through a 60-mesh sieve (250 *μ*m particle size). All dry dietary ingredients were homogenized finely before liquid oil was added. The configured mixture was passed through a 40-mesh sieve (425 *μ*m particle size). Finally, distilled water was added to the premixture (120 mL/kg dry ingredients). Dietary pellet was carried out to a 2.5 mm diameter by a cold press double helix plodder (CD4 ×1TS, SCUT, Guangdong, China). The scattered pellets were dried in a well-ventilated environment until the moisture was less than 10%. Pellets were labeled and stored in a refrigerator at -20°C.

### 2.3. Experimental Crayfish and Management Procedure

Female *C. quadricarinatus* adults were obtained from a breeding base in Chengmai, Hainan, China. Crayfish were acclimatized in a square pond (length × width × depth = 20 m × 18 m × 2 m) for one week before formal trial and feeding control diet. After acclimatization, crayfish (initial weight 72.32 ± 3.58 g) were randomly divided into four experimental treatments with quadruplicate net cages (1 m × 0.5 m × 1 m) at a density of 15 crayfish per cage that were hanging in the concrete pond. Polyvinyl chloride popes (15 cm length) were used to crayfish as shelters. Crayfish were hand-fed thrice daily at 09:00, 15:00, and 20:00 with a ratio of 6% wet body weight. Three hours after feeding, the uneaten diets were removed by siphon quickly. Cultivation water was exchanged at 60% every three days to maintain a normal and stable water quality environment. During the 10-week feeding trial, the water temperature was recorded as 24-27°C, the pH was recorded as 7.8-8.2, and the dissolved oxygen was maintained at >5.0 mg/L.

### 2.4. Sample Collection

After the 10-week feeding trial, crayfish in each cage were fasted for 24 h. After anesthetization in slurry ice, four crayfish were randomly taken from each replicate, and 1 mL disposable sterile syringes were used to sample hemolymph from the cardio coelom under carapace. Hemolymph were centrifuged at 3,500 rpm for 10 min at 4°C (3-18KS, Sigma, Germany). After centrifugation, liquid serum was separated into the centrifuge tubes and stored at -80°C. All the individuals, hepatopancreas, and ovaries were weighed to evaluate the gonadosomatic index (GSI) and hepatosomatic index (HSI). The formulas are listed as follows:

(1) $$\begin{array}{c} \text{Gonadosomatic index }\left(\text{GSI},\%\right)=\frac{\text{ovary tissue weight}}{\text{body weight}}\times 100,\\ \text{Hepatosomatic index }\left(\text{HSI},\%\right)=\frac{\text{hepatopancreas tissue weight}}{\text{body weight}}\times 100. \end{array}$$

The unilateral ovary tissue of six crayfish in each net cage was separated and fixed in 4% paraformaldehyde for the histology assay. Then, hepatopancreas, ovary, and eyestalk tissues were collected quickly on ice and frozen with liquid nitrogen immediately and stored at -80°C for further determination and analysis.

### 2.5. Paraffin Sections of the Ovary and Oocyte

The excised ovaries were fixed in paraformaldehyde (4%) for 24 h and then dehydrated in a gradient concentration of ethanol. The dehydrated ovarian tissues were then hyalinized in xylol and followed by embedding in paraffin. The embedded ovaries were sectioned with a rotary microtome at 5 *μ*m thickness. Ovarian slices were stained with the hematoxylin and eosin (H&E). Ovarian stained sections were observed by an optical microscope (ECLIPSE 200, Nikon, Japan). Image-Pro Plus 6.0 software was used to take digital images, and oocyte (late-stage yolk synthesis stage oocyte and mature oocyte) number and diameter were statistically analyzed by ImageJ software.

### 2.6. Lipid Metabolism, Antioxidative, and Initial Immune Health Assays

The hepatopancreas and ovary tissues were weighed and homogenized in precooled saline solution with 10-fold volumes (*v*/*w*). These homogenates were centrifuged at 3,500 rpm in 4°C for 10 min (3-18KS, Sigma, Germany) and the supernatants were collected. The supernatants of the hepatopancreas and ovaries and serum were diluted with 0.85% saline solution and follow the instruction and preexperiment for subsequent operations.

The supernatants of hepatopancreas and ovary homogenates were taken to determine the contents of triacylglycerol (TG) (glycerophosphate oxidase-peroxidase method) [23, 24] and total cholesterol (T-CHO) (cholesterol oxidase-peroxidase method) [25]. The concentrations of high-density lipoprotein cholesterol (HDL-C), low-density lipoprotein cholesterol (LDL-C) (enzymatic method) [26, 27], malondialdehyde (MDA) (thiobarbituric acid method) [28], glutathione peroxidase (GSH-Px) (5, 5′-dithiobis-(2-nitrobenzoic acid) method) [29], superoxide dismutase (SOD) (hydroxylamine method) [30], and total antioxidant capacity (T-AOC) (Fe^3+^ reduction method) [31] were detected to evaluate the antioxidant capacity of crayfish fed diets with different phospholipids. All detection was performed by the colorimetric approach of commercial reagent kits (Nanjing Jiancheng Bioengineering Institute, China). The measurements of these parameters were performed by the standard method, and the detection of optical density (OD) values was analyzed by a microplate reader (Epoch, BioTek, USA).

### 2.7. Gonad-Stimulating/Inhibiting Hormone Detection

The concentrations of gonad-stimulating/inhibiting hormones in the ovary and eyestalk were detected by enzyme-linked immunosorbent assay (ELISA) kits (Jianglai Biotechnology Co., Ltd., Shanghai, China). The supernatants of ovarian tissue homogenate were used to detect the concentrations of 17*β*-estradiol (E2), progesterone, methyl farnesoate (MF), and luteinizing hormone (LH) (antigen and antibody binding-chromogenic method). Eyestalks were also homogenized in prechilled 0.86% saline solution (10%, *w*/*v*) at a frequency of 65 Hz for 30 s at 4°C (Tissuelyser-24, Jingxin Technology, Shanghai, China). The homogenates were centrifuged at 3,500 rpm for 15 min at 4°C (3-18KS, Sigma, Germany). The supernatants of the eyestalk homogenate were collected to detect the concentrations of molt-inhibiting hormone (MIH) and gonad-inhibiting hormone (GIH) (antigen and antibody binding-chromogenic method). All tests were carried out according to the standard protocol.

### 2.8. Nontargeted Ovarian Lipidomic Analysis

#### 2.8.1. Metabolite Extraction

Ovarian tissue samples were separated with a wet weight of 20 mg and then homogenized after adding 400 *μ*L H_2_O. 200 *μ*L homogenate was absorbed and transferred to new tubes and then supplemented with H_2_O to 400 *μ*L. After mixing, 960 *μ*L of extract solution (methyl tert − butyl ether/methanol = 5 : 1) with internal standard was added. The mixtures were sonicated for 10 min in an ice-water bath after a 30 s vortex (YM-080S, Fangaowei Co., Ltd, China). Samples were centrifuged at 3,000 rpm for 15 min at 4°C (Heraeus Fresco 17, Thermo, USA) followed by transferring the supernatant (500 *μ*L) to a new tube. The following steps were performed twice: 500 *μ*L methyl tert-butyl ether was added to the mixture, which was then vortexed for 30 s, sonicated for 10 min, and centrifuged for 15 min at 3,000 rpm at 4°C, and 500 *μ*L supernatant was transferred to a new tube. Three copies of 500 *μ*L supernatant were collected and mixed for drying in a vacuum concentrator at 37°C (VOS-310C, EYELA, Japan). After drying, the samples were reconstituted in 200 *μ*L of 50% methanol in dichloromethane by sonication in ice water for 10 min. The mixtures were centrifuged at 13,000 rpm for 15 min, and then, 75 *μ*L of the supernatant was transferred to a fresh bottle for liquid chromatography-mass spectrometry/mass spectrometry (LC-MS/MS) analysis. Quality control (QC) samples were prepared by mixing equal aliquots of the supernatants from all of the experimental samples.

#### 2.8.2. LC-MS/MS Analysis

LC-MS/MS analysis was performed by using a Phenomenex Kinetex C18 column (2.1 × 100 mm, 1.7 *μ*m) coupled with TripleTOF 6600 MS (AB Sciex) using an ultra-high-performance liquid chromatography system (GC2010 Plus, Shimadzu, Japan). Mobile phase A consists of 60% acetonitrile, 40% water, and 10 mmol/L ammonium formate. Mobile phase B (10% acetonitrile and 90% isopropanol and 50mL 10mmol/L ammonium formate) was added per 1 L mixed solvent. The elution gradient was 0.0~12.0 min, 40%~100% B; 12.0~13.5 min, 100% B; 13.5~13.7 min, 100%~40% B; and 13.7~18.0 min, 40% B; column temperature is 45°C. The automatic injection temperature was 4°C, and the injection volumes were 0.5 *μ*L (positive) and 6 *μ*L (negative).

High resolution mass spectrometry data were obtained by triple TOF 6600 high resolution mass spectrometry through the IDA (information-dependent acquisition) mode. Under the IDA mode, the software (Analyst TF 1.7, AB Sciex) can select ions and collect the secondary MS data automatically according to the primary MS data and preset criteria. The top 12 ions with the strongest intensity greater than 100 were selected for secondary MS scanning in each cycle. The collision-induced dissociation energy was 30 eV, and the accumulation time of the secondary spectrum was 50 ms. The ion source parameters used in this detection were as follows: ion source 1: 40 psi, ion source 2: 80 psi, curtain gas: 25 psi, temperature: 650°C, ion spray voltage floating: 5,000 V (positive)/-4,000 V (negative), and declustering potential: 60 V.

#### 2.8.3. Data Preprocessing and Annotation

The original MS data were converted to a file in mzXML format using the converting software provided by ProteoWizard. Peak detection was first applied to the MS1 data. Then, the CentWave algorithm in the XCMS and MS/MS spectra was used for peak recognition, retention time correction, peak extraction, peak integration, and peak alignment. The minFARc was set as 0.6, and the cutoff value was set as 0.6. Lipid molecules were identified and qualified by using the lipid blast library through one-time spectrum matching. The absolute contents (ng/mg) of these lipid molecules were determined according to the SIL-IS and RF information. Partial least squares discriminant analysis (PLS-DA) and principal component analysis (PCA) were used to find and exclude outliers in each group of ovary samples to ensure high-quality repeatability and reliability of the data within the range. The difference between groups was defined by orthogonal partial least squares discriminant analysis (OPLS-DA). The model variable importance projection (VIP) > 1 and fold change (FC) > 1 (*P* < 0.05). The quantitative values of lipid metabolites were calculated by a Euclidean distance matrix.

### 2.9. Ovarian Transcriptome

Total RNA was extracted from ovary tissues of crayfish using TRIzol (R1100, Solarbio, China). An Agilent 2100 bioanalyzer (Agilent, Santa Clara, CA, USA) and a NanoDrop spectrophotometer (Thermo Fisher Scientific, Waltham, MA, USA) were used to accurately detect the RNA integrity, concentration, and quality. The starting RNA of library construction was total RNA. The first strand cDNA was synthesized in the MMuLV reverse transcriptase system. Fragmented mRNA was used as the template and random oligonucleotides were used as the primers. Then, the RNA strand was degraded by RNaseH. The second strand cDNA was synthesized with dNTPs as raw material in the DNA polymerase I system.

After purification, the double-stranded cDNA was repaired by terminal repair, adding a poly A tail to the sequencing joint. AMPure XP beads were used to screen 370-420 bp cDNA, which was then subjected to the PCR amplification. The PCR products should be purified again by AMPure XP beads, and then, the library was obtained. Following the construction of the library, preliminary quantification was accomplished by Qubit 2.0 Fluorometer. Then, the library was diluted to 1.5 ng/*μ*L. The insert size of the library was detected by an Agilent 2100 bioanalyzer. When the insert size satisfied the expectation, qPCR accurately quantified the effective concentration of the library to ensure the library quality (effective concentration was higher than 2 nM). After the library inspection was qualified, Illumina sequencing was performed after pooling different libraries according to the requirements of effective concentration and target offline data volume, and 150 bp paired-end readings were generated. Sequencing by synthesis is the basic principle for sequencing. Four dNTPs with fluorescent labels, adapter primers, and DNA polymerase were all added to the sequencing flow cell to accomplish the amplification reaction. If each sequencing cluster extends the complimentary chain, each addition of fluorescently labeled dNTPs will release the corresponding fluorescence signal. The sequencing instrument can capture the fluorescence signal and bioinformatics software can convert these light signals into sequencing peaks, which can obtain the sequence information of the measured fragment.

The image data of sequencing fragments measured by high-throughput sequencing instruments can be transformed into reads by CASAVA base recognition. To ensure the quality and reliability of the data analysis, the original data need to be filtered. It mainly includes removing reads with adapters, removing reads containing N (N means uncertain base information), and removing reads with low quality (reads with Qphred ≤ 20 bases accounting for more than 50% of the whole read length). Meanwhile, the Q20, Q30, and GC contents of the clean data were calculated. All subsequent analyses were of high quality based on clean data.

According to the KO and KEGG pathway annotation, the counts of unigenes under pathway hierarchy 2 were statistically analyzed and are shown in the form of bar charts. Clustering patterns of ovarian differential gene expression levels in crayfish under four different experimental diets were analyzed by the FPKM within the combinations, which was shown by a visualize heatmap. The read count samples from the gene expression levels were used for significant difference analysis (*q*-value < 0.005 and log2 fold change > 1) based on DESeq2 [32, 33]. Scatterplot maps were generated based on pairwise comparisons of the top 20 pathways enriched. The transcriptome libraries have been deposited into the NCBI SRA database with accession number PRJNA837726.

### 2.10. Statistical Analysis

In the present study, all data are expressed as the mean ± standard error (mean ± SE). Statistical analysis was performed using SPSS statistics 25.0 (SPSS Inc., Chicago, IL, USA). Statistical significance was determined using one-way analysis of variance (ANOVA) and Duncan's multiple range test. Among these results, *P* < 0.05 was considered to be statistically significant, and *P* < 0.01 was considered to be extremely significant. All figures in this study were drawn and layout using GraphPad Prism 7.0 (GraphPad Inc., San Diego, CA, USA).

## 3. Results

### 3.1. Hepatosomatic and Gonadosomatic Indexes of *C. quadricarinatus*

**Figure 1:**
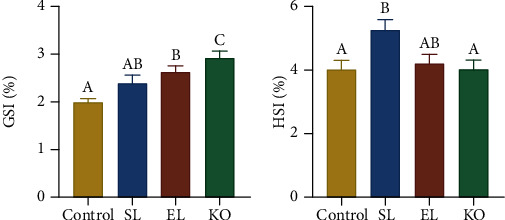
Hepatosomatic index and gonadosomatic index of *Cherax quadricarinatus* fed control, soybean lecithin (SL), egg yolk lecithin (EL), and krill oil (KO) diets for 10 weeks. (a) Gonadosomatic index. (b) Hepatosomatic index. Data are expressed as the mean ± SEM (standard error of the mean) (*n* = 20, individuals). Different uppercase letters (A, B, and C) indicate significant differences (*P* < 0.05) (C > B > A). HSI = hepatosomatic index; GSI = gonadosomatic index.

Dietary phospholipid supplementation significantly increased the GSI of crayfish (*P* < 0.05). Meanwhile, crayfish fed a diet with KO showed the maximum GSI among all the experimental treatments (Figure 1(a)). In contrast to GSI, crayfish fed a diet with SL had the highest HSI compared to the other experimental treatments (*P* < 0.05). No significant difference was found in the HSI of crayfish among the control, EL, and KO treatments (Figure 1(b)).

### 3.2. Ovarian Histology

**Figure 2:**
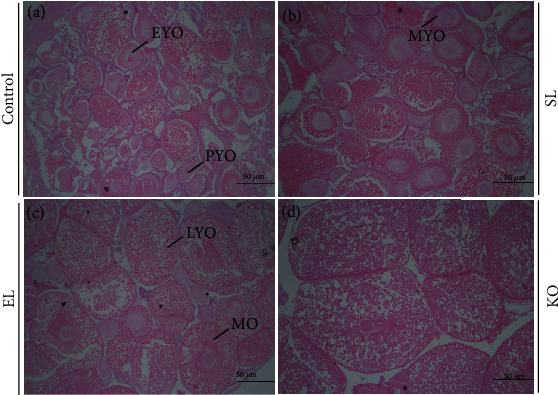
The ovarian histological characteristics of *C. quadricarinatus* fed four diets with different phospholipid sources for 10 weeks. Ovarian paraffin sections with H&E staining of *C. quadricarinatus* fed the control (a), SL (b), EL (c), and KO diets (d) with 50 *μ*m scale bars (40x magnification). PYO = preyolk synthesis stage oocyte; EYO = early-stage yolk synthesis stage oocyte; MYO = metaphase yolk synthesis stage oocyte; LYO = late-stage yolk synthesis stage oocyte; MO = mature oocyte.

In the same magnification field (40x) of ovary histology, significantly different developmental stages and diameters of oocytes can be observed (Figures 2(a)–2(d)). In the control, there were preyolk synthesis stage oocytes (PYO), early-stage yolk synthesis stage oocytes (EYO), metaphase yolk synthesis stage oocytes (MYO), late-stage yolk synthesis stage oocytes (LYO), and mature oocytes (MO) in the microscopic field. In the ovaries of crayfish fed a diet with SL, more LYO and MO were present than in the control. In the EL treatment, the oocyte diameter was larger than that in the control and SL. Among these four experimental treatments, crayfish fed the diet with KO had the largest MO.

The proportions and diameters of LYO and MO in the ovaries of crayfish fed diets with different phospholipid sources are shown in Table 2. By statistical analysis of all oocyte stages from all ovarian histological samples, dietary phospholipid supplementation significantly increased the proportions of LYO and MO (*P* < 0.05). This proportion in EL and KO was significantly higher than that in the control and SL (*P* < 0.05), and no significant difference was found between EL and KO. Crayfish fed SL and KO diets showed significantly higher average diameters of LYO and MO than those fed the control and EL diets (*P* < 0.05). Between the SL and KO treatments, there was no significant difference in the average diameter of the ovaries of crayfish.

### 3.3. Lipid Contents in Ovary, Hepatopancreas, and Serum

**Figure 3:**
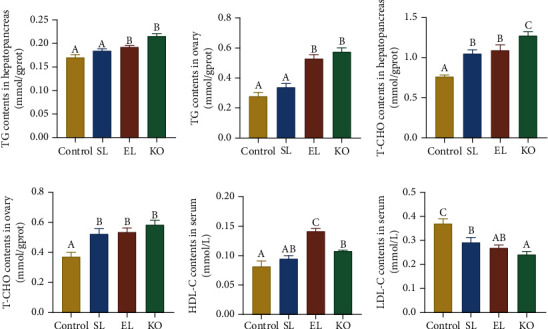
Lipid contents of *C. quadricarinatus* fed four diets with different phospholipid sources for 10 weeks. TG contents (a) and T-CHO contents (b) in hepatopancreas tissue. TG contents (c) and T-CHO contents (d) in ovary tissue. HDL-C contents (e) and LDL-C contents (f) in serum. Data are expressed as the mean ± SEM (standard error of the mean) (*n* = 4, replicate tanks). Different uppercase letters (A, B, and C) indicate significant differences (*P* < 0.05) (C > B > A). TG = triacylglycerol; T-CHO = total cholesterol; HDL-C = high-density lipoprotein cholesterol; LDL-C = low-density lipoprotein cholesterol.

Soybean lecithin supplementation did not increase the TG contents in either the hepatopancreas or ovary of crayfish compared to the control. EL and KO intake significantly increased the TG contents in these two key tissues (*P* < 0.05) (Figures 3(a) and 3(b)). Compared to TG, T-CHO contents in the hepatopancreas and ovary of crayfish fed phospholipid-supplemented diets were significantly higher than those in the control (*P* < 0.05). Crayfish fed KO showed the highest T-CHO level in the hepatopancreas among all experimental treatments (*P* < 0.05) (Figures 3(c) and 3(d)).

In serum, EL and KO supplementation in diets significantly improved the concentration of HDL-C compared with the control and SL diets, especially EL, which induced the highest content of HDL-C (*P* < 0.05) (Figure 3(e)). Phospholipid supplementation in diets significantly decreased the LDL-C contents in the serum of crayfish compared with the control (*P* < 0.05). Meanwhile, in crayfish fed the KO diet, the LDL-C content in serum was the lowest among all experimental treatments (Figure 3(f)).

### 3.4. Sex Hormone Concentrations in the Eyestalk and Ovary

**Figure 4:**
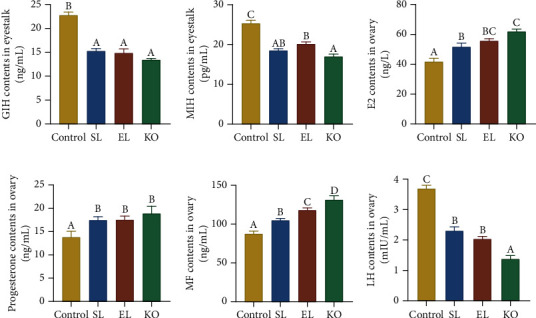
Gonad-inhibiting hormone and gonad-stimulating hormone concentrations in the eyestalk of *C. quadricarinatus* fed four diets with different phospholipid sources for 10 weeks. (a) Gonad-inhibiting hormone and (b) molt-inhibiting hormone concentration in the eyestalks. (c) 17*β*-Estradiol, (d) progesterone, (e) methyl farnesoate, and (f) luteinizing hormone concentrations in the ovary. Data are expressed as the mean ± SEM (standard error of the mean) (*n* = 4, replicate tanks). Different uppercase letters (A, B, and C) indicate significant differences (*P* < 0.05) (C > B > A). GIH = gonad-inhibiting hormone; MIH = molt-inhibiting hormone; E2 = 17*β*-estradiol; MF = methyl farnesoate; LH = luteinizing hormone.

Phospholipid supplementation in diets significantly decreased the concentrations of GIH and MIH secreted by the eyestalk of crayfish (*P* < 0.05) (Figures 4(a) and 4(b)). There was no significant difference in the GIH contents of crayfish fed the SL, EL, and KO diets. Crayfish fed the KO diet showed significantly lower MIH content than those fed the EL diet (*P* < 0.05).

In ovary tissue, dietary phospholipids significantly increased the concentrations of E2, progesterone, and MF compared with the control (*P* < 0.05), which received no extra phospholipids (Figures 4(c)–4(e)). KO diet intake achieved the highest E2 and MF concentrations in the ovaries of crayfish compared to other experimental diets (*P* < 0.05). There was no significant difference in the concentrations of progesterone in the ovary among the SL, EL, and KO dietary treatments. Phospholipid supplementation in diets significantly decreased the concentrations of LH in the ovaries of crayfish (*P* < 0.05) (Figure 4(f)). There was no significant difference in the LH contents of crayfish fed the SL and EL diets. The lowest LH content in the ovary was found in crayfish fed the KO diet.

### 3.5. Antioxidative and Innate Immune Status

**Figure 5:**
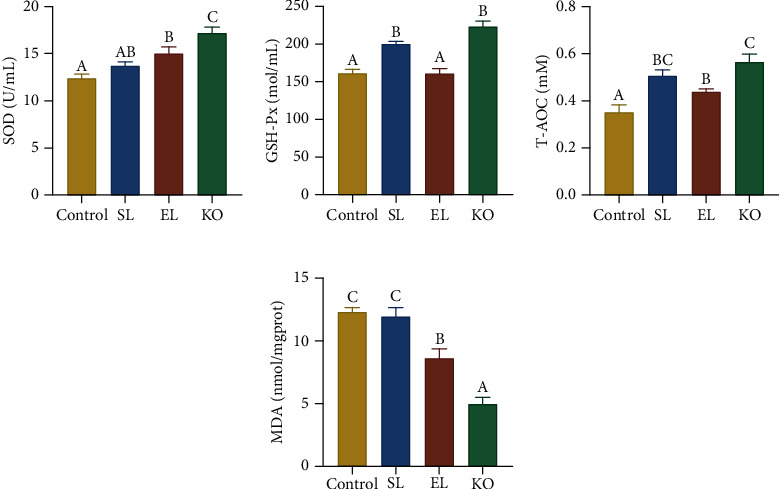
Antioxidant capacity of *C. quadricarinatus* fed four diets with different phospholipid sources for 10 weeks. (a) Superoxide dismutase activity. (b) Glutathione peroxidase activity. (c) Total antioxidant capacity. (d) Malondialdehyde content. Data are expressed as the mean ± SEM (standard error of the mean) (*n* = 4, replicate tanks). Different uppercase letters (A, B, and C) indicate significant differences (*P* < 0.05) (C > B > A). SOD = superoxide dismutase; GSH-Px = glutathione peroxidase; T-AOC = total antioxidant capacity; MDA = malondialdehyde.

Crayfish fed the EL and KO diets showed significantly higher SOD activities than those fed the control diet (*P* < 0.05) (Figure 5(a)). SL and KO diets significantly increased the activities of GSH-Px in serum compared with the control and EL treatments (*P* < 0.05) (Figure 5(b)). Similarly, the SL and KO diets also significantly increased the T-AOC of crayfish compared with the control and EL diets (*P* < 0.05) (Figure 5(c)). There were significantly lower MDA contents in the serum of crayfish fed the EL and KO diets than in the control and SL diets (*P* < 0.05). There was no significant difference in the MDA contents of crayfish fed the control and SL diets (Figure 5(d)).

### 3.6. Ovarian Lipidomics

**Figure 6:**
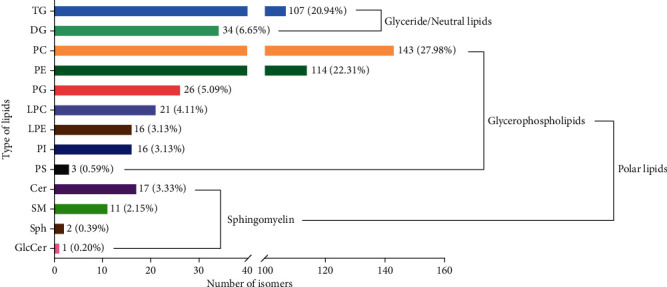
Lipid subclass types, isomer numbers, and percentages identified in the ovaries of *C. quadricarinatus* fed diets with different phospholipid sources for 10 weeks. TG = triacylglycerol; DG = diacylglycerol; PC = phosphatidylcholine; PE = phosphatidylethanolamine; PG = phosphatidylglycerol; LPC = lysophosphatidylcholine; LPE = lysophosphatidylcholine; PI = phosphatidylinositol; PS = phosphatidylserine; Cer = ceramide; SM = sphingomyelins; Sph = sphingosine; GlcCer = glycosylceramide.

The classification of target lipids detected and identified in ovaries of crayfish fed four diets containing different phospholipids was further investigated through ultra-high-performance liquid tandem chromatography quadrupole time of flight mass spectrometry (UHPLC-QTOFMS). In total, 511 lipid species were detected in the ovarian tissue of crayfish assigned to 13 lipid classes. The 13 lipid classes in decreasing order were 143 phosphatidylcholines (PCs) (27.98%), 114 phosphatidylethanolamines (PEs) (22.31%), 107 triacylglycerols (TGs) (20.94%), 34 diacylglycerols (DGs) (6.65%), 26 phosphatidylglycerols (PGs) (5.09%), 21 lysophosphatidylcholines (LPCs) (4.11%), 17 ceramides (Cers) (3.33%), 16 lysophosphatidylethanolamines (LPEs) (3.13%), 16 phosphatidylinositols (PIs) (3.13%), 11 sphingomyelins (SMs) (2.15%), 3 phosphatidylserines (PSs) (0.59%), 2 sphingosines (Sphs) (0.39%), and 1 glycosylceramides (GlcCers) (0.20%) (Figure 6).

**Figure 7:**
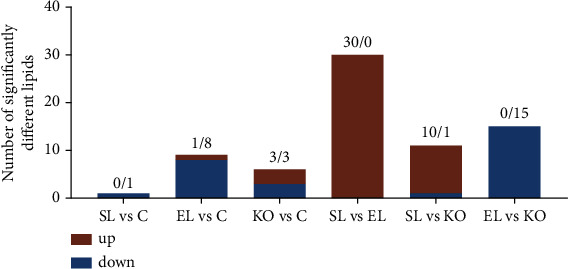
Number of significantly changed lipid molecules in the ovary of *C. quadricarinatus* fed diets with different phospholipid sources for 10 weeks.

Compared to the control, there were 1, 8, and 3 downregulated lipid molecules in the ovaries of crayfish fed SL, EL, and KO, respectively. There were 1 and 3 upregulated lipid molecules in the ovaries of crayfish fed the EL and KO diets, respectively. Compared to EL, there were 30 increased lipid molecules in crayfish fed SL, such as PC (20:1/22:6), PE (19:0/18:2), and TG (20:5/18:2/18:1). Compared to KO, 10 increased and 1 decreased lipid molecules were observed in crayfish fed the SL diet, and 15 decreased lipid molecules were found in crayfish fed the EL diet, such as PC (22:1/18:3), PE (40:9), TG (22:6/20:5/20:5), and TG (22:0/18:3/16:0). (Figure 7). Heatmaps were used to show the significantly different lipid molecules in ovaries between crayfish fed two experimental diets. Specific lipid molecules of significantly different contents are shown in Figure S1. PC, PE, and TG were the three most abundant significantly different lipid molecules in the ovaries of crayfish between the two experimental diets. The percentage ratio of TG, PC, PE, PS, and LPE in the whole lipids in ovary of *C. quadricarinatus* fed four experimental diets are shown in Figure S2.

### 3.7. Ovarian Transcriptome

**Figure 8:**
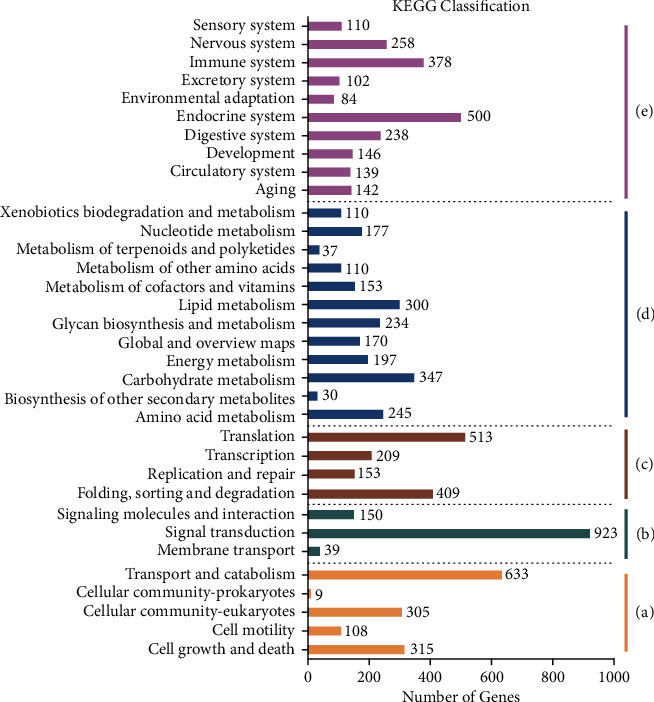
Highly expressed biological pathways represented in the KEGG database of *C. quadricarinatus* fed diets with different phospholipid sources for 10 weeks. a = cellular processes; b = environmental information processing; c = genetic information processing; d = metabolism; e = organismal systems.

Through comparison with the KEGG database, 34 KEGG pathways were identified that belonged to 5 pathway hierarchy1 categories associated with cellular processes, environmental information processing, genetic information processing, metabolism, and organismal systems (Figure 8). Among these pathways, “signal transduction,” “translation,” “transport and catabolism,” and “endocrine system” were all significantly enriched and were involved in more than 500 DEGs. The “cell growth and death,” “cellular community-eukaryotes,” “folding, sorting, and degradation,” “transcription,” “amino acid metabolism,” “carbohydrate metabolism,” “glycan biosynthesis and metabolism,” “lipid metabolism,” “digestive system,” “immune system,” and “nervous system” pathways were all enriched and involved in more than 200 DEGs.

**Figure 9:**
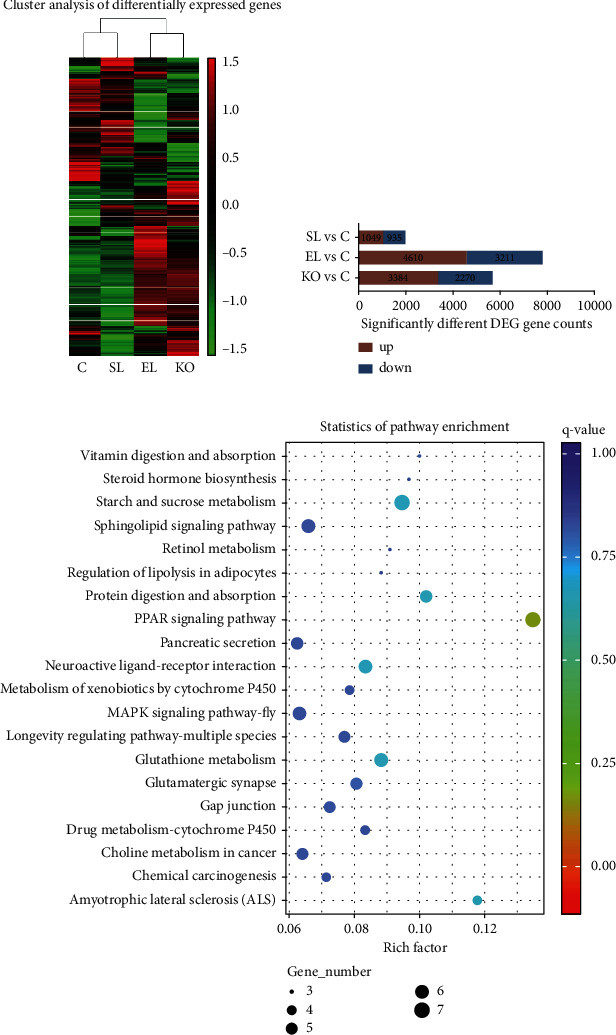
Clustering heatmap analysis of differentially expressed genes in the ovaries of *C. quadricarinatus* among the four experimental groups (a). Number of significantly changed gene counts in the ovaries of *C. quadricarinatus* between the three experimental groups and the control (b). Scatterplot for pathways in KEGG enrichment of differentially expressed genes in the ovary of *C. quadricarinatus* between the KO and EL groups (c).

Compared to the control, there were 1,049 significantly upregulated genes and 935 significantly downregulated genes in crayfish fed the SL diet. In crayfish fed the EL diet, 4,610 genes were upregulated significantly and 3,211 genes were downregulated significantly compared to the control. In crayfish fed the KO diet, 3,384 genes were increased, and 2,270 genes were decreased expression significantly compared to the control (Figure 9(b)). By hierarchical clustering genes that were significantly different in the ovaries of crayfish fed four experimental diets, the control and SL groups showed a similar pattern and clustered together. Meanwhile, EL and KO showed a similar pattern and had the same cluster (Figure 9(a)). According to the similarity of the EL and KO treatments, compared to EL, there were significantly changed pathways in the ovaries of crayfish fed the KO diet (Figure 9(c)). Among them, “vitamin digestion and absorption,” “steroid hormone biosynthesis,” “starch and sucrose metabolism,” “retinol metabolism,” “regulation of lipolysis in adipocytes,” “protein digestion and absorption,” “pancreatic secretion,” and “glutathione metabolism” were all involved.

## 4. Discussion

Lipid supplementation in the diet is crucial for organisms in terms of cellular functions, such as signaling and transport, and the activity of membrane-bound enzymes, which are quite beneficial to animal health [9]. For female individuals, phospholipids are also necessary for the development of the ovary, embryo, and initial larval stages [34]. Especially in crustaceans, yolk (including amounts of lipids and proteins) needs to provide sufficient nutrients to the embryo and larva until start feeding. In this study, SL, EL, and KO supplementation in diets significantly increased the GSI of *C. quadricarinatus* compared to the control. In contrast to EL and KO, SL diet intake can significantly improve the HSI of crayfish. This result indicated that animal phospholipid sources are superior to plant phospholipids in promoting ovarian development in crayfish. A similar result was found in Chinese mitten crab (*Eriocheir sinensis*), which showed that phospholipid supplementation in diets can significantly improve GSI, and a better effect was found in diets with animal phospholipids [22]. In rainbow trout (*Oncorhynchus mykiss*), 6% EL in the diet can significantly improve the survival of fry, but SL cannot [35]. In naturally, occurring phospholipids, unsaturated FAs are predominantly located in the sn-2 position, and saturated FAs are predominantly connected to the sn-1 position. Compared to plant phospholipids, animal phospholipids, especially marine animal phospholipids, contain more PUFAs, such as eicosapentaenoic acid (EPA) and docosahexaenoic acid (DHA) [9]. Therefore, crayfish fed the EL and KO diets showed better ovarian development status. From the ovarian transcriptome results, significantly changed genes within the column of crayfish fed the EL and KO diets were clustered together and crayfish fed the control and SL diets were clustered together. This clustering reflected the significant difference between animal phospholipid and plant phospholipid digestion, catabolism, and utilization by crayfish during ovarian development.

As a kind of lipid, phospholipids can not only provide essential nutrients but also promote the transport and deposition of lipids and FAs by emulsification in crustaceans [36]. During ovary maturation of crustaceans, phospholipids can also promote lipid transfer from the periapical hepatopancreas to the gonad [37]. In this study, phospholipid supplementation in diets significantly increased the T-CHO content in both the hepatopancreas and ovary of crayfish. Only animal phospholipid significantly improved the TG content in these two tissues compared to the control, and KO showed the most significant promotion. In eggs of *C. quadricarinatus*, proteins are the most abundant component (approximately 63.2%) followed by lipids (approximately 32.3%). With the prolongation of development, the lipid content decreased gradually and then was depleted, which indicated that lipids are the main energy source for the eggs of *C. quadricarinatus* [38]. As the primary location of energetic lipid storage, the hepatopancreas is the most important heterosynthetic tissue transferring lipids to gonads during gametogenesis and ovarian maturation in crustaceans [1]. Therefore, adequate lipid accumulation in the ovary and hepatopancreas can provide nutritional guarantees for the development of embryos and the metamorphosis of larvae. Although crustaceans are incapable of de novo synthesis of cholesterol, it is the most abundant type among all sterols in organisms and is also an essential component of lipoproteins and precursors of ecdysteroids and sesquiterpenoids [39]. Dietary supplementation with phospholipid improves lipid metabolism, including cholesterol metabolism by enhancing digestion, absorption, transport, and acyl donors. Therefore, in the study, T-CHO contents in ovary were significantly increased after phospholipids were ingested by crayfish regardless of their raw material.

The maturation of oocytes in crayfish ovaries is asynchronous. From the histology of ovaries of crayfish in the control, there are growing oocytes under different cellular stages and sizes. With the continuous transfer and accumulation of nutrients (mainly proteins, lipids, and carbohydrates), the proportions of LYO and MO in ovarian tissue were increased [20]. Phospholipid supplementation in diets can significantly accelerate this process and induce a higher ratio of LYO and MO in ovary of crayfish. In the ovaries of crayfish fed the KO diet, abundant yolk globule deposition in LYO and MO induced oocyte proliferation and larger diameters than those in the control and EL treatments. Referred to as yolk proteins in crustaceans, lipovitellin is different from that in vertebrates, which lack protein phosphates and show a high content of lipids [40]. As the precursor of lipovitellin, vitellogenin (Vg) is a crucial transporter of lipids from the hemolymph to female gonadal tissue during vitellogenesis. Previous research indicated that female specific Vg is one of the high-density lipoproteins (HDLs) and is also the main protein fraction in the ovary of crustaceans, and lipid transport can be realized by combining with HDL [41]. In the present study, two kinds of animal lipids both increased the contents of HDL in crayfish, and the highest value was found in the EL treatment due to the rich concentration of carotenoids in egg yolk [42]. Lipovitellin of crustaceans characteristically contains a variety of carotenoids, including astaxanthin, *β*-carotene, canthaxanthin, and other intermediary metabolites. HDL is also frequently associated with carotenoids and is usually referred to as lipovitellin [43].

Low-density lipoproteins (LDLs) are closely related to cholesterol metabolism in crustaceans [44]. Although they are incapable of synthesizing cholesterol de novo and must be absorbed from dietary nutrition, cholesterol is the essential precursor of steroidal hormone biosynthesis [39]. As the ovaries mature, cholesterol is converted to steroidal hormones that separate from LDL and cause a decrease in LDL cholesterol in the serum of crayfish. Compared to the control, female crayfish fed diets supplemented with SL, EL, and KO showed significantly higher stimulatory sex hormone concentrations in ovary tissue. Compared to EL, crayfish fed the KO diet also showed a significantly enriched KEGG pathway of “steroid hormone biosynthesis.” Due to their low molecular weight and lipid solubility, these hormones can be transported by Vg through the hemolymph and stored within oocytes as conjugates of lipovitellin, which regulates ovarian maturation and embryogenesis [45]. 17*β*-Estradiol (E2) and progesterone are two typical vertebrate steroids that have been formally present and play key roles in gonadal development in a variety of crustaceans [46]. Injection *in vivo* and incubation *in vitro* with estrogen and progesterone can both improve Vg synthesis and oocyte development status in female crustaceans, including freshwater crayfish [46–48]. Methyl farnesoate (MF) is a kind of naturally occurring sesquiterpenoid synthesized by the mandibular organ and belongs to the juvenile hormone (JH) family in insects [49]. *In vivo*, MF concentrations have been correlated with ovarian maturation and vitellogenesis in the crab (*O. senex senex*), red swamp crayfish (*Procambarus clarkii*), and *C. quadricarinatus* [50–52]. In contrast to the significantly increased contents of E2 and progesterone, luteinizing hormone (LH) concentrations in the ovary were significantly decreased when crayfish were fed diets with phospholipids. Although LH plays a crucial role in controlling ovarian development and final oocyte maturation in fish [53], feedback regulation among several gonadal steroidal hormones within the hypothalamus-hypophyseal axis still exists. In mammals, the secretion of LH can be negatively regulated by ovarian steroid hormones, including E2 and progesterone, and the nature of these influences is time- and dose-dependent [54].

Crustacean reproductive behavior and ability are regulated by the neurosecretory system. In the KEGG classification results, five hundred genes were involved within the endocrine system in the ovary after ingestion of diets with SL, EL, and KO. Traditionally, eyestalk ablation is the most commonly used endocrine manipulation to induce gonadic maturation and spawning in a variety of crustaceans. The removal of the X organ sinus gland complex in the eyestalk can significantly weaken the obstruction of gonadal development due to the secretion of inhibiting hormones [39]. GIH and MIH are two principal inhibitory neuropeptides secreted by the optic ganglia that play crucial roles in the regulation of reproduction and molting in crustaceans [45, 55]. In the present study, phospholipid supplementation in diets not only significantly decreased the concentrations of GIH and MIH in eyestalk tissue but also increased the concentrations of promoting sex hormones in ovary tissue of *C. quadricarinatus*. Therefore, compared to eyestalk ablation, crucial nutrient provision (such as phospholipids) in the diet can be a more humane strategy for promoting ovary development and maturation in economic crayfish by reducing inhibiting hormones and enhancing promoting hormones.

There are different compositions among the three phospholipid sources, and krill oil has a significant advantage in improving antioxidant capacity. In this study, crayfish fed the KO diet showed the lowest MDA content and the highest activities of SOD, GSH-Px, and T-AOC in the hepatopancreas. In addition to phospholipids, there are relatively high percentages of PUFAs and antioxidants, such as astaxanthin, in KO. Evidence indicates that diets with PUFAs, especially if there is an imbalanced ratio between n-3 and n-6 PUFAs, impose oxidative damage in organisms [56]. However, in the study, such oxidative damage did not occur but showed stronger antioxidant protection in crayfish fed the KO diet. On the one hand, phospholipid in KO can improve the bioavailability of lipid components including PUFAs [57]. On the other hand, this positive effect can be attributed to the high amounts of astaxanthin ingredients in krill oil, which are carotenoids. Carotenoids cannot be synthesized by animals endogenously and need to ingest these natural substances from daily feeding. In humans, astaxanthin has several key physiological beneficial functions, including inhibition of PUFA oxidation in cell membranes and modulation of exacerbated inflammatory responses [58, 59]. Meanwhile, the combination of astaxanthin and fish oil, which is rich in n-3 PUFAs, can surpass an excess benefit due to an additional antioxidant protection provided at the catecholaminergic-rich anterior forebrain in rats [60].

Compared to EL, crayfish fed the KO diet showed more positive antioxidant capacity, which was also present in the ovarian transcriptome results. Glutathione metabolism was significantly enriched with significantly increased mRNA expression of glutathione S transferase (GST), spermine synthase, and glutamate-cysteine ligase catalytic subunit. This result indicated that GSH/GST antioxidant systems were activated and contributed. GSH is a tripeptide composed of glutamate, cysteine, and glycine that is quite important in protecting organelles from the toxification induced by ROS. As a multifunctional detoxification enzyme, GST can catalyze the conjugate reaction of the sulfhydryl group within GSH to several electrophiles [61]. At the molecular level, the binding of nuclear factor erythroid 2-related factor 2 (Nrf2) to antioxidant responsive elements (AREs) can trigger the transcription of antioxidant genes including SOD, GSH-Px, GST, and GSH biosynthetic enzymes [62]. In coronary heart disease patients, Antarctic krill oil treatment can activate antioxidant signaling Nrf2 in peripheral blood leukocytes [63]. Additionally, in rats, treatment with KO significantly increased splenic and hepatic Nrf2 mRNA expression and GSH content when compared to the iron group [64].

Compared to the control, the number of significantly changed genes in ovary of crayfish fed the diet with EL or KO was higher than that in crayfish fed the diet with SL. As discussed above, animal phospholipids showed better positive effects on ovarian development in *C. quadricarinatus*. In these two animal phospholipids, KO ingestion by crayfish significantly enriched the KEGG pathways of “retinol pathway”, and “vitamin digestion and absorption” and focused on retinol dehydrogenase, cubilin, and biotinidase. In mammals, retinol is essential for female reproduction, and retinoids possess crucial physiological functions in ovarian steroidogenesis, oocyte maturation, and formation of the corpus luteum [65]. The rate-limiting conversion of retinol to retinal, which is the precursor of retinoic acid, is performed by retinol dehydrogenases [66]. Meanwhile, retinol is a lipid-soluble vitamin also known as vitamin A. A higher ratio of TG to PUFAs in KO can supply sufficient carriers for the transport of lipid-soluble vitamins including retinol. This can be verified by the significantly enriched lipid metabolism pathways and significantly increased gene expression of pancreatic triacylglycerol lipase in ovaries of crayfish fed the KO diet compared with those fed the EL diet. As a water-soluble vitamin, the synthesis of biotin and the absorption of vitamin B12 were both significantly improved by the ingestion of KO, which is reflected in the changes in biotinidase and cubilin. Previous research indicated that biotin was incorporated into the follicles in growing female ovarian follicles, especially in a larger amount immediately before the ovulation stage. Meanwhile, biotin is a necessary substance for embryonic development and should be supplemented and utilized immediately after fertilization [67]. In conclusion, for a more holistic presentation, the experimental summary is shown in Figure S3.

## 5. Conclusion

A compound diet with phospholipid supplementation can significantly improve gonadal development quality in *Cherax quadricarinatus*. Krill oil can be an optimum choice for female reproductive *C. quadricarinatus* by enhancing the secretion of gonad-stimulating hormones, absorption and metabolism of vitamins, and deposition of lipid and yolk granules during ovarian development. Due to the difference in composition among the three phospholipids, krill oil can also improve antioxidant capacity and effectively alleviate the potential lipid peroxidation damage caused by the active metabolism of PUFAs in *C. quadricarinatus*.

## Figures and Tables

**Table 1:** Ingredient formulation (g/kg dry matter) and analyzed proximate composition (%) of the four practical experimental diets with different phospholipids fed to female *Cherax quadricarinatus*.

Item	Experimental diets			
	Control	SL	EL	KO
Fish meal	290	290	290	290
Soybean meal	190	190	190	190
Cottonseed	190	190	190	190
Corn starch	100	100	100	100
Wheat flour	100	100	100	100
Soybean oil	50	30	30	30
Cholesterol^1^	10	10	10	10
Soybean lecithin^2^	0	20	0	0
Egg yolk lecithin^3^	0	0	20	0
Krill oil^4^	0	0	0	20
Vitamin premix^5^	10	10	10	10
Mineral premix^6^	10	10	10	10
Ca(H_2_PO_4_)_2_	10	10	10	10
Choline chloride	10	10	10	10
Sodium carboxymethyl cellulose	30	30	30	30
Total	1000	1000	1000	1000

Analyzed proximate composition (%)				
Moisture	9.46	9.43	9.62	9.22
Crude protein	43.65	43.49	43.47	43.58
Crude lipid	14.27	14.30	14.27	14.59
Ash	11.25	11.19	11.40	11.62

^1^Purchased from Sangon Biotech, Ltd., Shanghai, China. ^2^Purchased from Shanghai Taiwei, Ltd., Shanghai, China. ^3^Purchased from Shanghai Taiwei, Ltd., Shanghai, China. ^4^Purchased from Qingdao Kangjing Marine Biotech, Ltd., Qingdao, China. ^5^Reference as Chen et al., 2020. ^6^Reference as Chen et al., 2020.

**Table 2:** The proportion and average diameter of late-stage yolk synthesis stage oocytes (LYO) and mature oocyte (MO) in *Cherax quadricarinatus* fed four diets with different phospholipid sources for 10 weeks.

Parameters of LYO and MO	Experimental diets			
	Control	SL	EL	KO
Proportion	40.00 ± 2.27^a^	52.25 ± 2.17^b^	62.75 ± 2.53^c^	65.25 ± 2.49^c^
Average diameter	58.21 ± 2.38^a^	70.87 ± 2.79^b^	63.29 ± 1.56^a^	76.25 ± 2.17^b^

Note: data are expressed as the mean ± SEM (standard error of the mean) (*n* = 20, individuals). Different lowercase letters (a, b, and c) indicate significant differences (*P* < 0.05) (c > b > a).

## Data Availability

All the data can be obtained in the manuscript and supplementary materials.

## Supplementary Materials

Supplementary Materials The lipid molecules and clustering heatmap of lipid molecules in the ovary of *C. quadricarinatus* fed four experimental diets are shown in Figure S1. The percentage ratio of TG, PC, PE, PS, and LPE in the whole lipids in the ovary of *C. quadricarinatus* fed four experimental diets is shown in Figure S2. For a more holistic presentation, the experimental summary is shown in Figure S3.
