# Supplementary material for: Evaluation of the Role of Soybean Lecithin, Egg Yolk Lecithin, and Krill Oil in Promoting Ovarian Development in the Female Redclaw Crayfish Cherax quadricarinatus
Source: Aquac Nutr. 2023 Jan 12;2023:6925320. doi: 10.1155/2023/6925320 (PMC9973198; doi:10.1155/2023/6925320)
Supplement: Supplementary Materials — The lipid molecules and clustering heatmap of lipid molecules in the ovary of C. quadricarinatus fed four experimental diets are shown in Figure S1. The percentage ratio of TG, PC, PE, PS, and LPE in the whole lipids in the ovary of C. quadricarinatus fed four experimental diets is shown in Figure S2. For a more holistic presentation, the experimental summary is shown in Figure S3. [file 6925320.f1.docx]

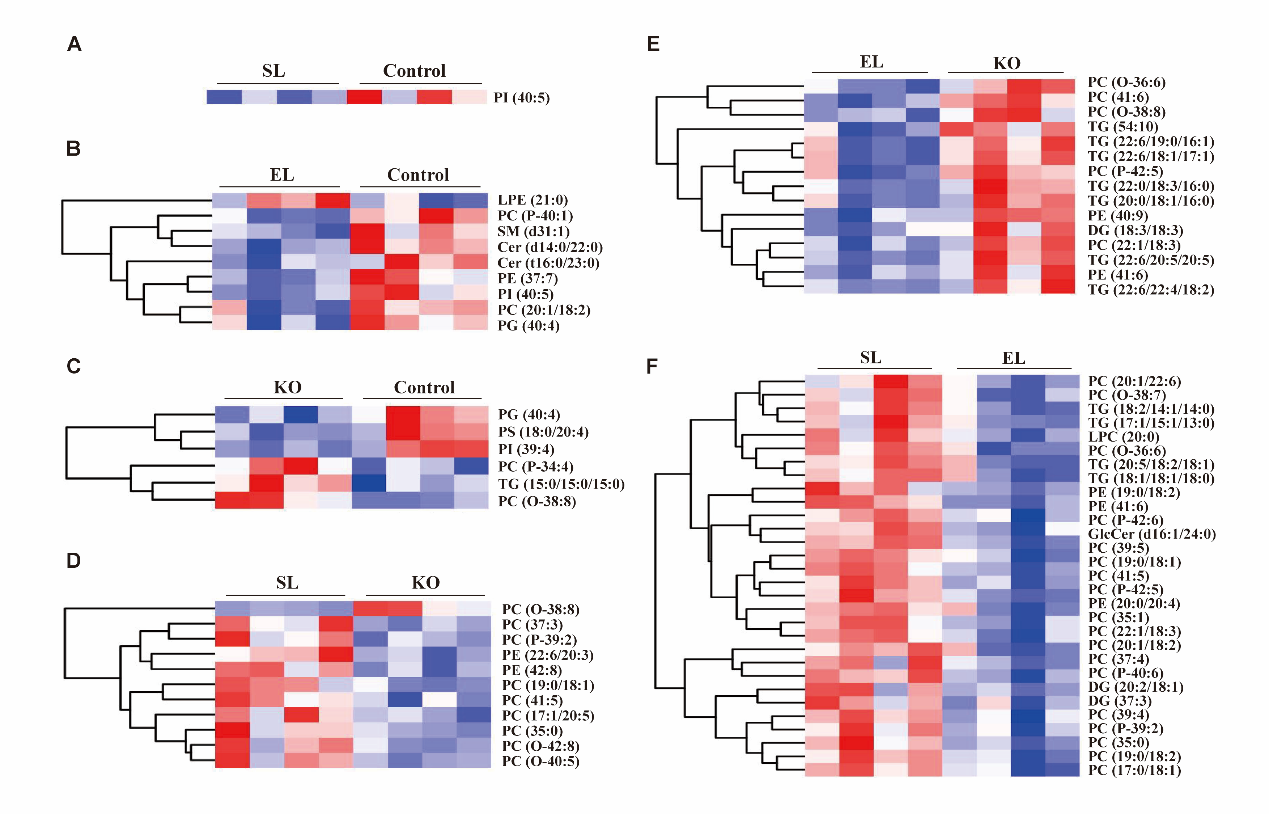


**Figure S1**

Figure legend: The lipid molecules and clustering heatmap of lipid molecules in the ovaries of *C. quadricarinatus* fed diets with different phospholipid sources for 10 weeks


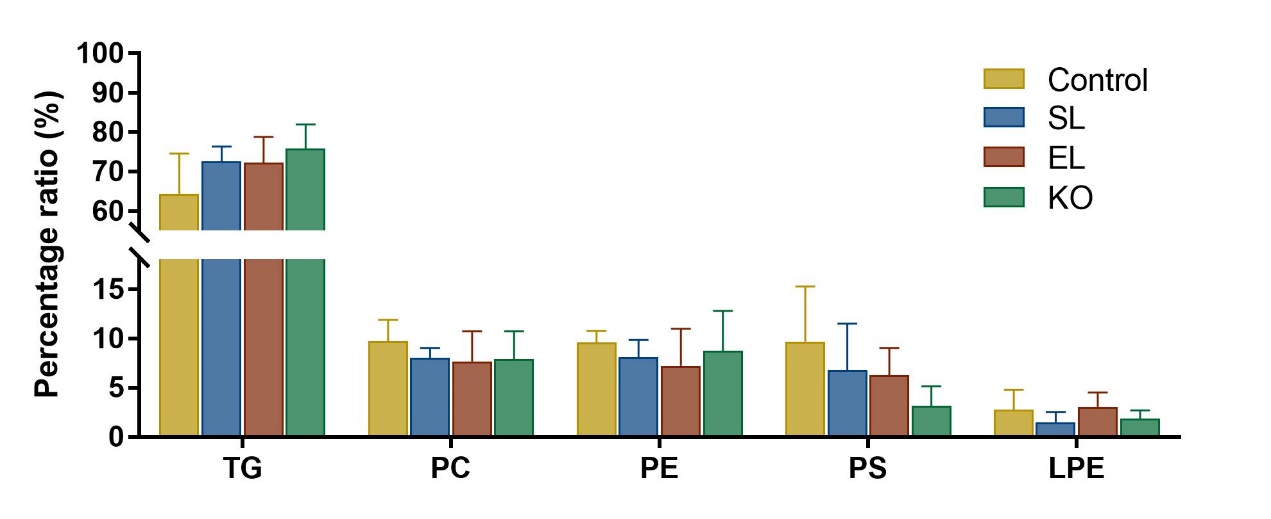


**Figure S2**

Figure legend: The percentage ratio of TG, PC, PE, PS and LPE in the whole lipids in ovary of *C. quadricarinatus* fed four diets with different phospholipid sources for 10 weeks.


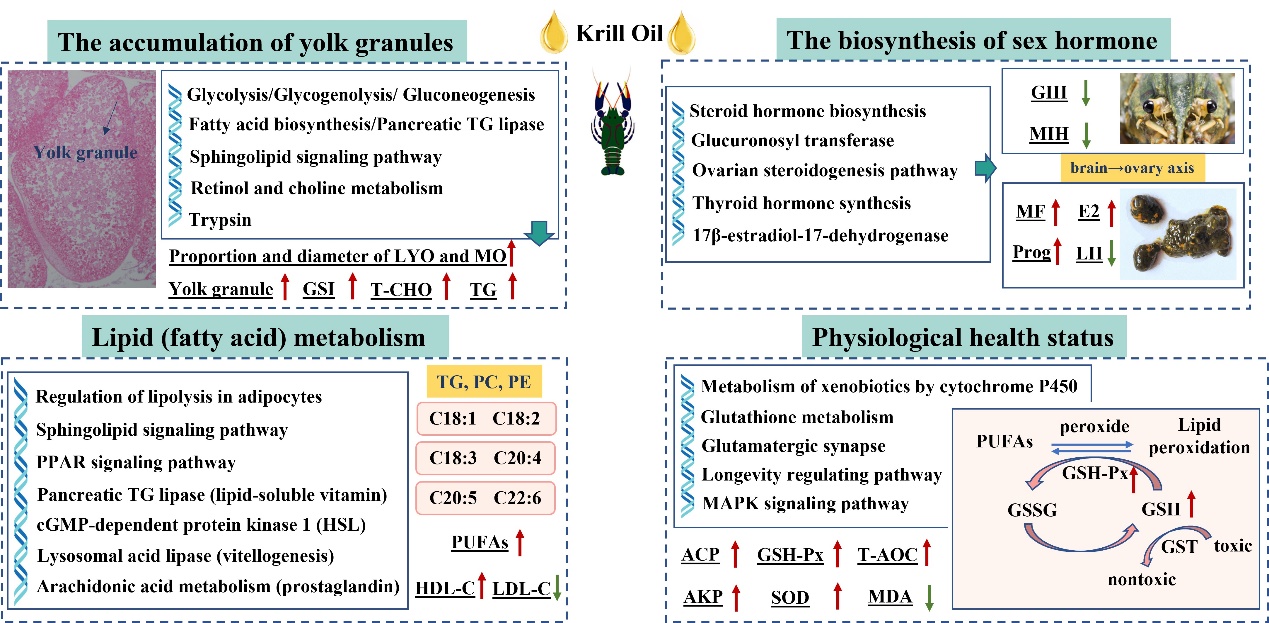


**Figure S3**

Figure legend: Summary of metabolic response of *C. quadricarinatus* fed diet with krill oil.
